# Predicting potential distribution of poorly known species with small database: the case of four‐horned antelope *Tetracerus quadricornis* on the Indian subcontinent

**DOI:** 10.1002/ece3.2037

**Published:** 2016-03-04

**Authors:** Krishna Prasad Pokharel, Tobias Ludwig, Ilse Storch

**Affiliations:** ^1^ Chair of Wildlife Ecology and ManagementUniversity of FreiburgTennenbacher Str. 4D – 79106FreiburgGermany

**Keywords:** Distribution range, endemic species, four‐horned antelope, Indian subcontinent, isolation

## Abstract

Information gaps on the distribution of data deficient and rare species such as four‐horned antelope (FHA) in Nepal may impair their conservation. We aimed to empirically predict the distribution of FHA in Nepal with the help of data from the Indian subcontinent. Additionally, we wanted to identify core areas and gaps within the reported range limits and to assess the degree of isolation of known Nepalese populations from the main distribution areas in India. The tropical part of the Indian subcontinent (65°–90° eastern longitude, 5°–30° northern latitude), that is, the areas south of the Himalayan Mountains. Using MaxEnt and accounting for sampling bias, we developed predictive distribution models from environmental and topographical variables, and known presence locations of the study species in India and Nepal. We address and discuss the use of target group vs. random background. The prediction map reveals a disjunct distribution of FHA with core areas in the tropical parts of central to southern–western India. At the scale of the Indian subcontinent, suitable FHA habitat area in Nepal was small. The Indo‐Gangetic Plain isolates Nepalese from the Indian FHA populations, but the distribution area extends further south than proposed by the current IUCN map. A low to intermediate temperature seasonality as well as low precipitation during the dry and warm season contributed most to the prediction of FHA distribution. The predicted distribution maps confirm other FHA range maps but also indicate that suitable areas exist south of the known range. Results further highlight that small populations in the Nepalese Terai Arc are isolated from the Indian core distribution and therefore might be under high extinction risk.

## Introduction

An important challenge in conservation biology is to understand the factors that determine the spatial distribution and abundance of species (Johnson 1980). With increasingly intensive human exploitation of land, habitats of wildlife species are being fragmented, degraded, and lost (Ellis et al. 2010; Haddad et al. 2015). Therefore, it becomes more and more important to understand the distribution of species over large spatial scale extents to guide targeted conservation management (Porwal et al. 1996; Mathys et al. 2006; Araújo et al. 2011). Human pressure on wildlife habitats is particularly high in developing parts of the world with large human populations, such as the Indian subcontinent (Mishra 1997; Sekhar 1998; Goswami et al. 2014). Predictive habitat modeling has been widely used as a tool to assess the impact of climate, land use, and environmental change on the distribution of organisms (Guisan and Zimmermann 2000; Drew et al. 2011). Such modeling approaches are also important for predicting potential species distribution ranges from environmental factors and for setting conservation priorities (Guisan and Thuiller 2005). A concise species distribution model (SDM) can inform conservation planning and management in a cost‐effective way to approach the conservation challenge (Sanderson et al. 2002).

**Figure 1:**
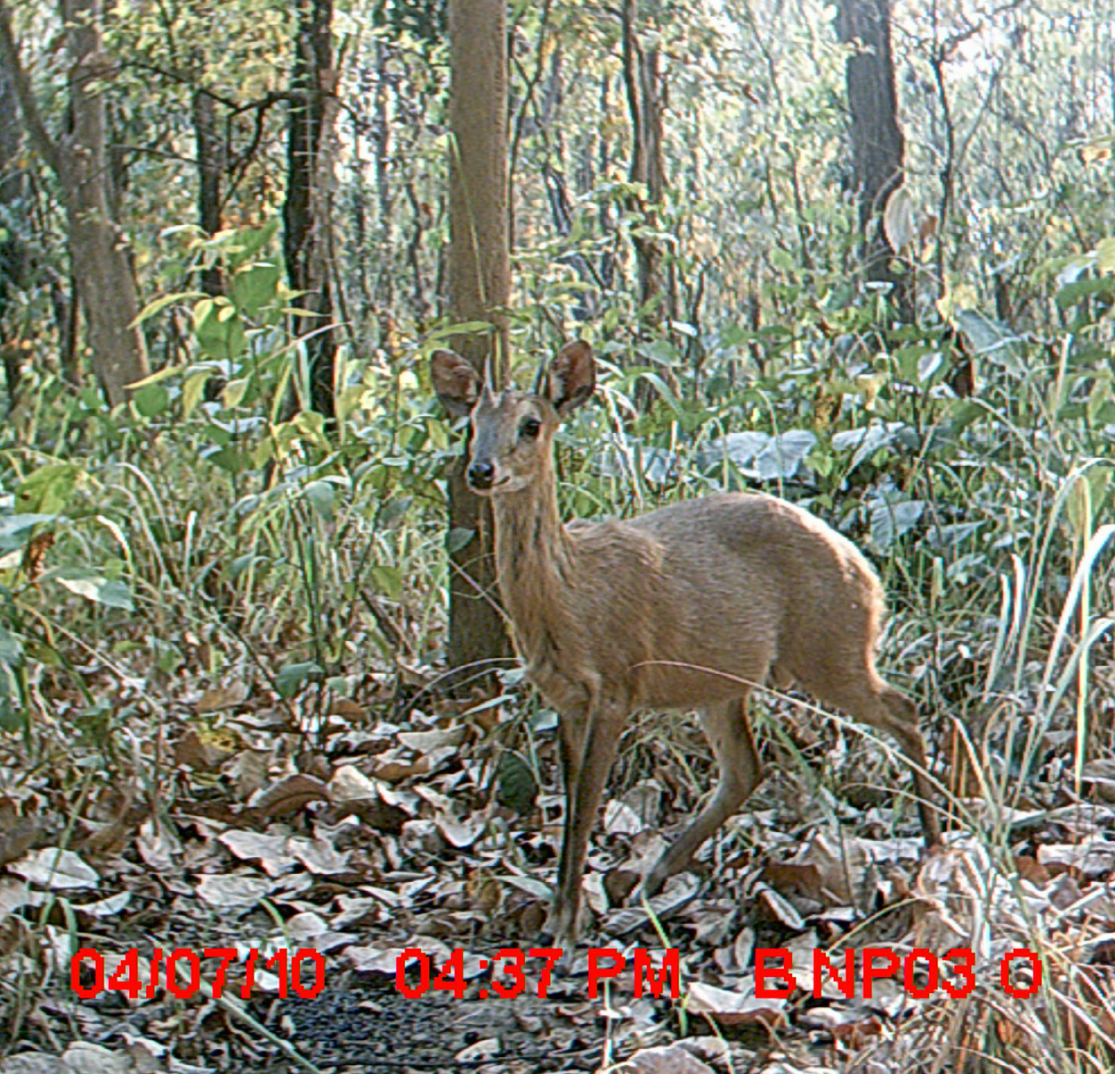
An adult male four‐horned antelope trapped in motion‐sensor cameras (stealth cam STC‐1550, model no. D‐40, USA) in the Sal *Shorea robusta* forest of Bardia National Park, Nepal (325 m elev.). (Image courtesy: KPP and the Department of National Parks and Wildlife Conservation, Nepal).

Four‐horned antelope (*Tetracerus quadricornis* de Blainville, 1816), hereafter “FHA” (Fig. 1), is endemic to the Indian subcontinent, that is, Nepal and India. It is a solitary herbivore with small body size (shoulder height 55–65 cm and weigh 18–21 kg at adult) (Karanth and Sunquist 1992; Leslie and Sharma 2009) and low density – less than 1 individual/km^2^ (Baskaran et al. 2009). It is classified as Vulnerable in the IUCN Red List of Threatened Species (Mallon 2008) and as data deficient in the national red list of Nepal (Jnawali et al. 2011). As the deforestation rate of the Indian subcontinent is still in the range of 1.5% to 2.7% per year (Puyravaud et al. 2010; Southworth et al. 2012), habitat destruction because of conversion to agricultural land is the major threat to this species. FHA is believed to be widely distributed with fragmented populations particularly in dry deciduous forest in lowland of Nepal and India (Krishna et al. 2008; Mallon 2008; Leslie and Sharma 2009; Sharma et al. 2013). Studies at local scale extents suggest that FHA occurrence is determined by tree species richness (Sharma et al. 2013) and their nutrient levels (Ahrestani et al. 2011). However, beside local explanations of the species–habitat relationships (Krishna et al. 2008; Baskaran et al. 2009; Sharma et al. 2013; Pokharel et al. 2015), a comprehensive and empirical assessment of the FHA's distribution range is still lacking (Krishna et al. 2009; Leslie and Sharma 2009).

**Figure 2:**
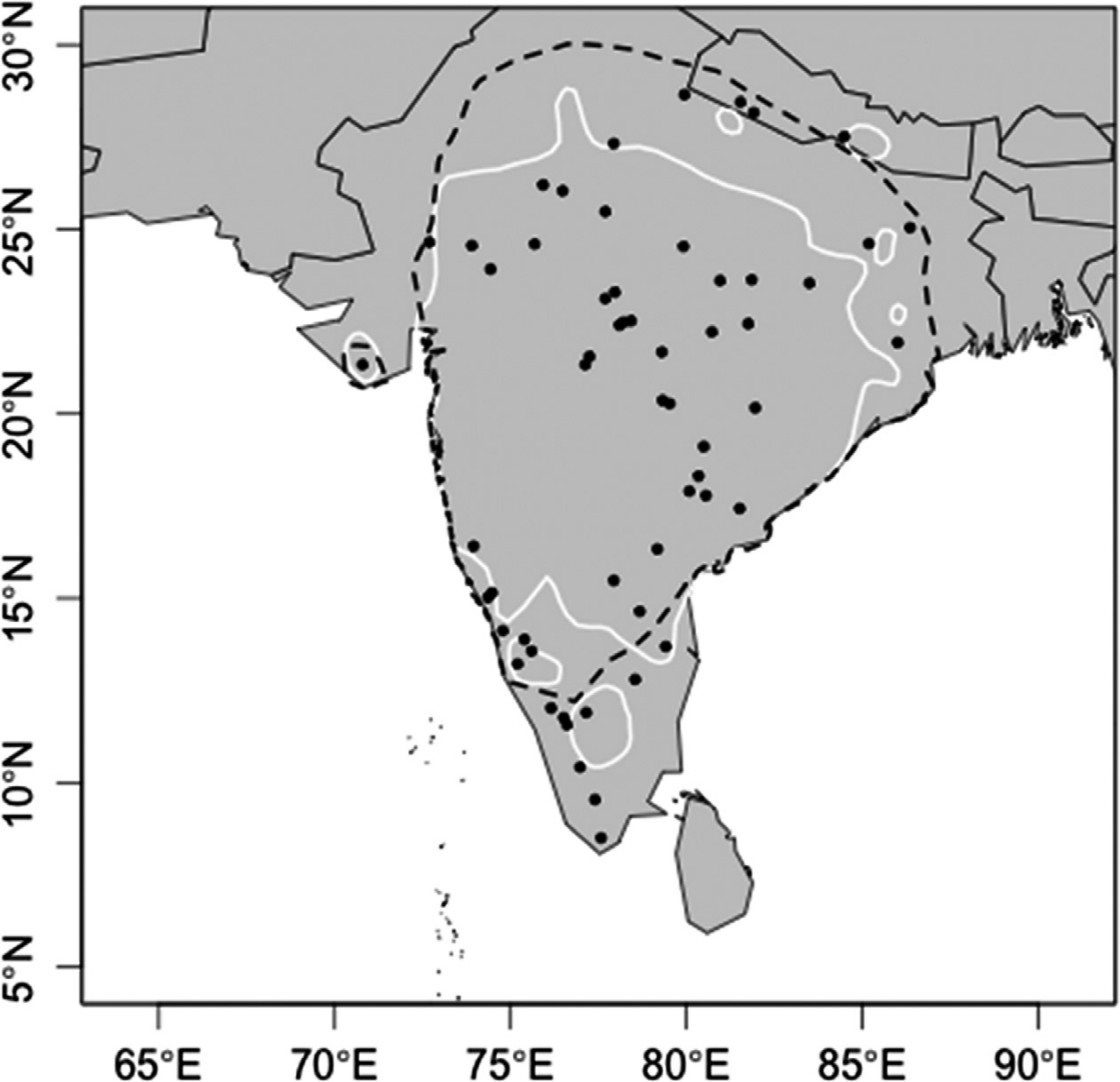
Distribution range of four‐horned antelope on the Indian subcontinent according to 1) the IUCN Red List (Mallon 2008; delimited with hatched border) and 2) Leslie and Sharma (2009; delimited with a white border). Black points indicate the occurrence records used in this paper (see Methods for details).

According to Leslie and Sharma (2009), the species occurs in east central Nepal, whereas the IUCN Red List predicts FHA to occur only in the western part of Nepal (Fig. 2). Own observations, however, confirm FHA to be present in both Chitwan and Bardia (28°23′0″N, 81°30′0″E)/ Banke (28°11′28″N 81°54′46″E) national parks west of it. Figure 2 reveals some more discrepancies between range maps and occurrence locations, especially in the southernmost part of India. Missing information about the ecology and distribution of this endemic species may impair its conservation, particularly in Nepal where the species is listed as data deficient. The main objective of this study therefore was to obtain a better impression about the potential distribution of FHA in Nepal and its isolation from the Indian core distribution. We also aimed to develop an empirically based map of the entire possible distribution range. We hypothesized that the Indo‐Gangetic Plain which is known as “bread basket” of South Asia (Aggarwal et al. 2004) isolates the suitable FHA habitats in Nepal from those in India. Furthermore, as FHA is restricted to dry deciduous forest in tropical regions (Krishna et al. 2008; Baskaran et al. 2009; Sharma et al. 2013; Pokharel et al. 2015), we expected that bioclimatic variables, which are related to a savannah‐like vegetation, can explain the distribution of FHA. We believe that the findings of this study will fill the knowledge gap regarding the distribution and status of the species; in particular, our findings will be helpful to ground truth potential occurrences, and to assess habitat conditions. Ultimately, conservation management can take actions for the conservation of FHA throughout their distribution range, particularly in the Nepalese lowland.

## Methods

### Study area

We focus on the tropical part of the Indian subcontinent (65°–90° eastern longitude, 5°–30° northern latitude) (Fig. 2), that is, the areas south of the Himalayan Mountains, particularly India (3 287 260 km^2^) and Nepal (147 180 km^2^) (data source: www.data.un.org accessed on 27 November 2014). Siwalik Hills (or Churia range) demarcate the northern limit of these tropical areas whereas the Indo‐Gangetic Plains (Ganges, Indus and Brahmaputra river valleys), Deccan plateau, and Western and Eastern Ghats are the main topographical features. Eastern coast and Western Ghats of India are dominated by humid climate and tropical evergreen or moist deciduous forests whereas in the western and northwestern part of India, dryness increases with longer (5–9 months) dry periods. Those areas, except Rajasthan, where thorny thickets dominate, are mainly dominated by dry deciduous forest (Blasco et al. 1996). Nepalese tropical forest and northern, northeastern, and central Indian forests are dominated by sal (*Shroea robusta*) (Blasco et al. 1996; Barnekow Lillesø et al. 2005; Carpenter 2005). More than two‐third of the tropical forest in this region is occupied by moist and dry deciduous forests, which frequently face wild fires (FAO, 2007; Joseph et al. 2009) and are the main potential habitat for FHA.

Most (~80%) of the precipitation in the subcontinent occurs due to monsoon from May to September are higher in the southeast of the subcontinent whereas westerly circulation derive some precipitation from November to March, and is more active in northwest (Mooley and Parthasarathy 1983; Shrestha et al. 2000; Duan and Yao 2004). Mean annual rainfall is about 1100 mm (1090.4 mm ± 103.91 mm) for India (Parthasarathy et al. 1994) and about 1800 mm for Nepal (Shrestha 2000). Mean temperature of the coldest months is generally >15°C in the tropical areas of the subcontinent.

### Species and environmental data preparation

We used a dataset of ungulate occurrence in 76 large (>200 km²) protected areas within India as provided in Ahrestani et al. (2011) and cross‐checked its coordinates in a GIS environment (ArcGIS 10.1, Redlands California) with two datasets of protected areas (WCU‐UNEP, 2007; IUCN & UNEP‐WCMC, 2015). We corrected one location from Ahrestani et al. (2011), which showed obvious latitudinal shift of 135 kilometers. All other coordinate pairs were inside the respective shapefiles from the protected areas databases. Thus, we extracted 53 locations with FHA presence. We added one additional location from Anwar et al. (2011) and three from Nepalese protected areas: “Bardia,” “Banke,” and “Chitwan,” which are also known to harbor FHA (Pokharel et al. 2015; http://www.dnpwc.gov.np assessed on 2 December 2015). Thus, we obtained 57 FHA records.

Single coordinate pairs as representatives of large protected areas may introduce uncertainty into analysis. However, we considered location error to be low, relative to study area extent (3000 × 3000 km). For example, protected area minimum and maximum sizes were 259 km² and 7506 km², respectively, with a mean of 828 km². These area extents correspond to quadrats of 16, 87, and 29 km edge length. Assuming these squares on average to contain correct locations, minimum, maximum, and mean location errors relative to edge length of the total extent were 0.53%, 2.90%, and 0.97%, respectively. Given further the inherent spatial autocorrelation in environmental (bioclimatic) variables, we considered the given coordinate pairs to well reflect the underlying bioclimatic and topographic values.

We used 19 bioclimatic variables (Hijmans et al. 2005) to capture environmental variation within the potential distribution range of FHA. The set of 19 bioclimatic rasters was accessed from the WORLDCLIM website (http://www.worldclim.org/) at a resolution of 0.5 min (30 arc seconds, 0.083 degree, ca. 1 by 1 km) for tile twenty‐eight. As elevation was a good predictor of FHA occurrence at smaller scale extents (Pokharel et al. 2015), we complemented our set of predictors with elevation, based on a Shuttle Radar Topography Mission (SRTM) tile (USGS 2006). All raster layers were downloaded at a resolution of 0.083 degree (~1 km), georeferenced to geographic coordinate system (GCS WGS84), and cropped to 65°–90° eastern longitude and 5°–30° northern latitude for further use in a raster stack in RStudio (RStudio Team, 2015).

### Modeling procedure

We used maximum entropy (MaxEnt) modeling (Phillips et al. 2006), a machine learning approach for data analysis and prediction of FHA distribution. This method uses background points to describe the location of species presences in environmental space. It estimates an optimal probability distribution of maximum entropy (Phillips et al. 2006) and can be regarded as a niche‐based machine learning technique (Elith et al. 2011), which characterizes an approximation of a species’ ecological niche and projects it into geographic space. MaxEnt has been found to perform best among many different species distribution modeling methods particularly if available information is incomplete and sample size is small (Elith et al. 2006; Pearson et al. 2007). Similar to linear combinations in statistical models like GLMs and GAMs, MaxEnt uses an expanded set of transformations of the covariates, called features in machine learning techniques (Elith et al. 2011) and thus allows for flexibility with the predictors. However, it has recently been demonstrated that more complex features are not the main determinants of model performance (Syfert et al. 2013). We therefore used quadratic and hinge features and did not change the regularization parameter also because we were using a set of preselected variables as suggested by Merow et al. (2013), thus providing enough control for over‐fitting. We made use of MaxEnt's jackknife test, which delivers the effect of removal of each predictor variable on the increase of explanatory ability (gain) of the model (Elith et al. 2006), and to reduce the number of predictors until we reached the maximum possible gain. This procedure resulted in eight predictor variables for the final FHA distribution model (Table 1).

**Table 1:** Bioclimatic and topographical predictors and estimates of their relative contributions in the final model of FHA distribution on the Indian subcontinent

Variable	Description	Percent contribution	Permutation importance
bio17	Precipitation of driest quarter	22.7	26.3
bio4	Temperature seasonality	21.6	11.3
bio5	Max temperature of warmest month	19	21.5
bio9	Mean temperature of driest quarter	12.8	8.8
elev	Elevation	12.4	22.4
bio11	Mean temperature of coldest quarter	5.1	0.3
bio14	Precipitation of driest month	4.2	6.7
bio18	Precipitation of warmest quarter	1.5	2.7

One of the central issues in the presence‐only modeling is the choice of background values (Merow et al. 2013), because a random selection can lead to models that predict sampling effort rather than a species’ distribution. A way to account for sampling bias is to use a target group background, which has the same spatial bias as the occurrence data (Phillips et al. 2009). We selected Indian ungulates presented in Ahrestani et al. (2011) as a target group. Our background locations thus consisted of all 76 coordinates therein plus a set of 182 locations for 14 ungulates beside FHA, which we downloaded from the Global Biodiversity Information Facility (GBIF Backbone Taxonomy, 1 July 2013. accessed via http://www.gbif.org on 16 November 2014). After removal of duplicate records and addition of background locations for Nepal (Jnawali et al. 2011), we obtained 248 target group background locations.

We performed threshold‐independent evaluation with the receiver operating area under curve (AUC). We used MaxEnts inbuilt bootstrapping option to obtain a standard deviation for the area under the receiver operating curve (AUC) and to visualize uncertainty in species response curves. Bootstrapping is a nonparametric approach to statistical inference that can provide accurate inferences when sample size is small (Freedman 1981). Its implementation in MaxEnt selects training data by sampling with replacement from the presence points, with the number of samples equaling the total number of presence points. With this procedure, about one‐third of the presences are left out while all others are included between one and four times. Therefore, the training datasets contain duplicate records. We bootstrapped 100 times for calculation of AUC standard deviations and response curves. We prepared a final prediction map and compared our model predictions with FHA distribution ranges published in Leslie and Sharma (2009) as well as in the IUCN Red List of Threatened Species (Mallon 2008).

For a further assessment of model calibration, we derived presence‐only calibration (POC) plots (Phillips and Elith 2010) from model output. While AUC gives relative measures of discrimination ability, model calibration plots return how well predicted values match probabilities at occurrence and background locations. All analyses and raster processing were accomplished in R version 3.0 (R Core Team, 2013) with the dismo package, version 1.0–12 (Hijmans et al. 2015) and its dependencies.

## Results

### Model performance

**Figure 3:**
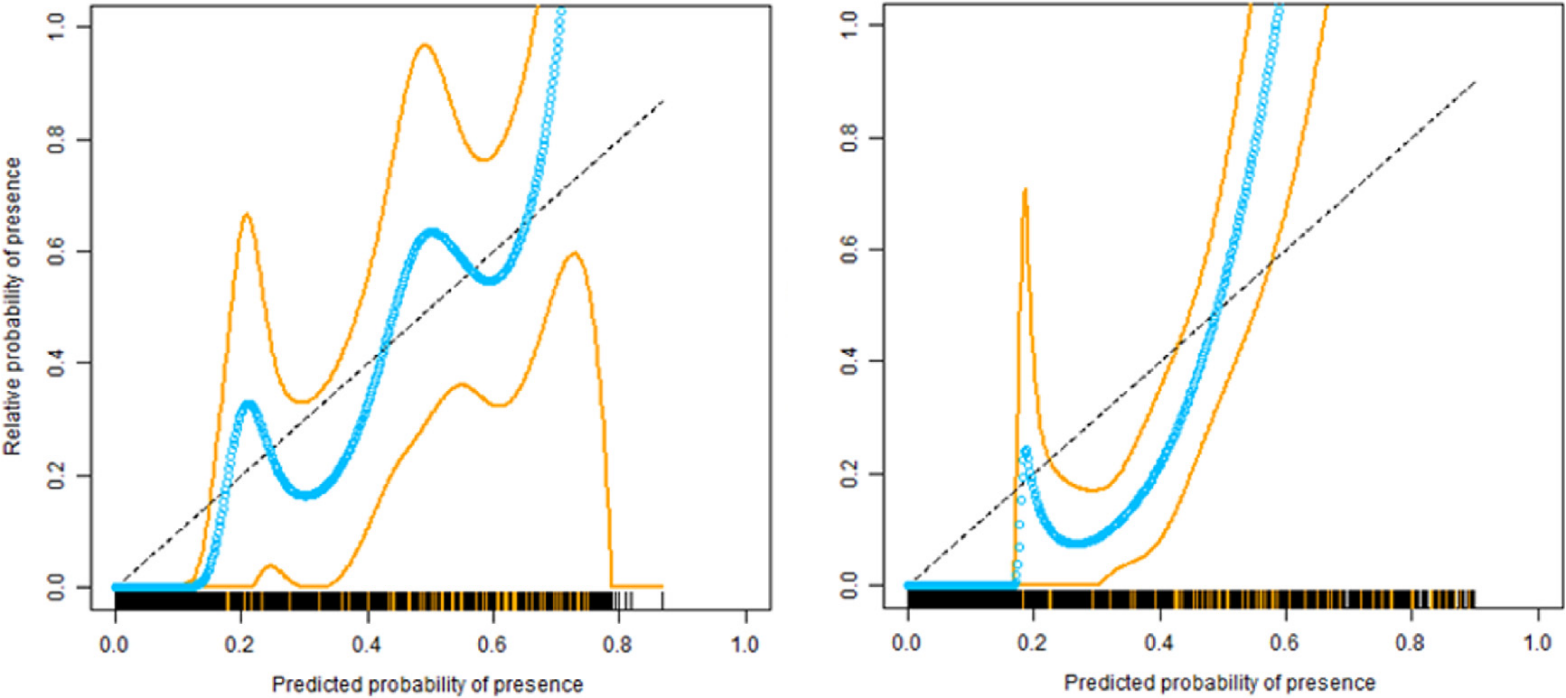
Presence‐only calibration (POC) plots for a model with target group background (left) and random background (right). The target group background model displays better calibration as it follows the diagonal of a perfectly calibrated model.

The area under receiver operating curve (AUC) obtained with 100 bootstraps was 0.86 ± 0.18 (AUC ± standard deviation) for the model with target group background and 0.92 ± 0.013 for a model with random background. POC plots revealed a better fit between predictions and relative probability of presence in the model that used target group background. Specifically, predicted probabilities of occurrence were allocated along the diagonals of calibration plot, whereas the model with random background was badly calibrated at prediction values >0.5 (Fig. 3).

### FHA distribution range

**Figure 4:**
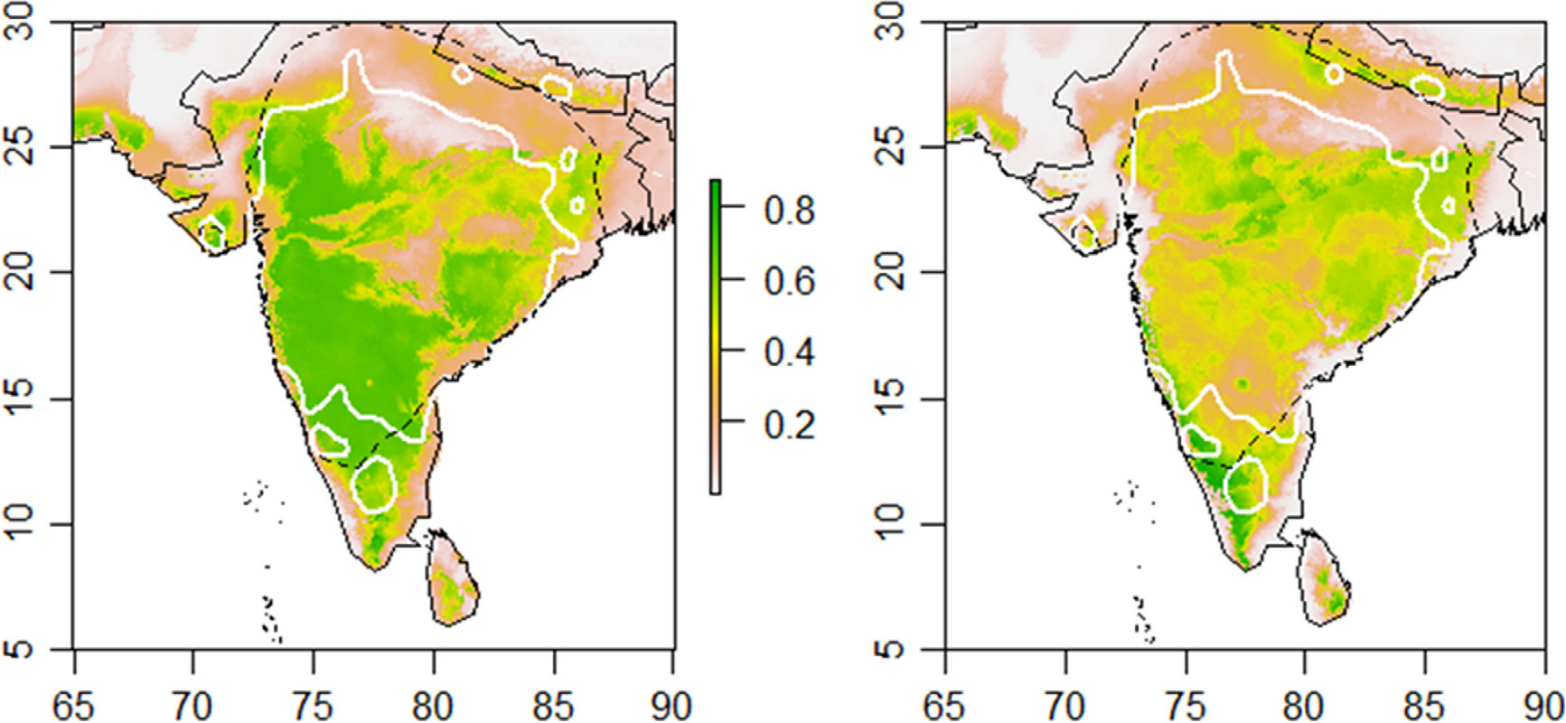
Predicted distributions for FHA on the Indian subcontinent showing the mean suitability score for a model with target group background (left) and random background (right). Hatched and white borders as well as axis units are the same as in Figure 2.

**Figure 5:**
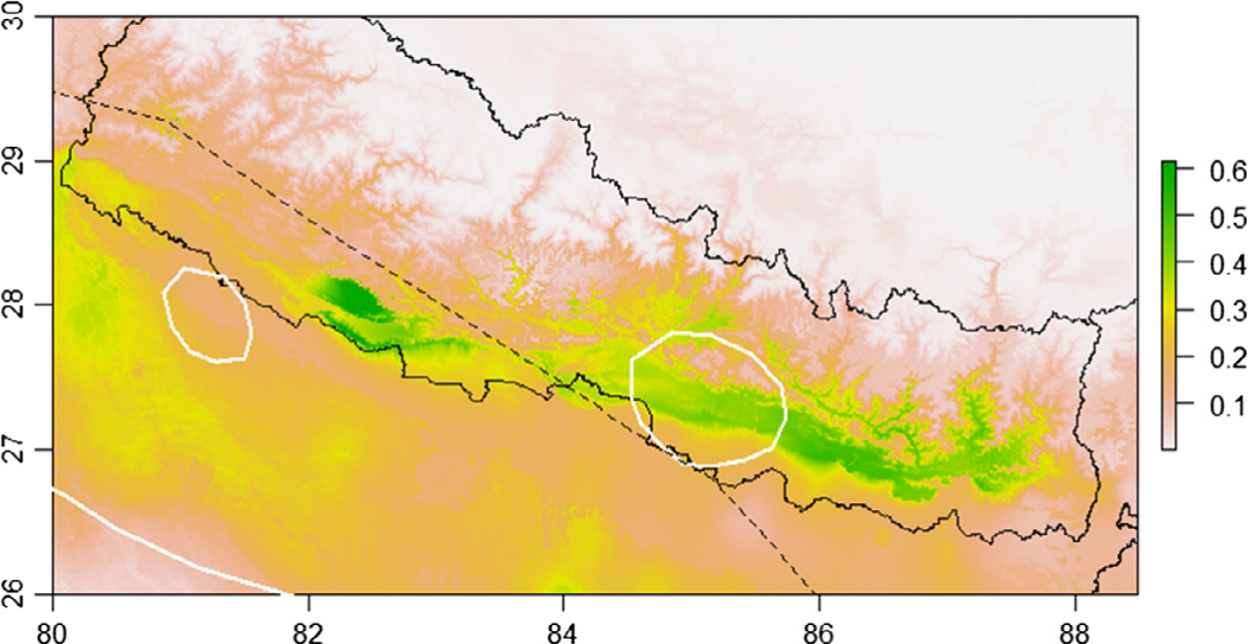
Predicted distribution of suitable FHA habitat in Nepal zoomed in from Figure 4 (left).

Large parts of India were found to be suitable for FHA (Fig. 4). The probability of occurrence clearly diminished toward the Indian northwest (Thar Desert) and was also lower in the Eastern Ghats, and very low in the Ganges river valley between India and Nepal. The Gangetic Plains had the lowest predicted suitability also without inclusion of the human land use predictor (results not shown). Furthermore, suitable areas along the Terai Arc in Nepal stretch along the narrow strip of Churia range that is fragmented within (Fig. 5). In particular, areas suitable for FHA in Nepal, that is, Chure hills in Sindhuli, Udayapur, and Dhanusha districts in east, Chitwan in center, and Dang and Banke in west Nepal, are poorly connected. In addition, areas predicted to be suitable for FHA lie in Gujarat state of India and the southern parts of Sindh Province in Pakistan (Fig. 4).

### Predictors of the FHA distribution

**Figure 6:**
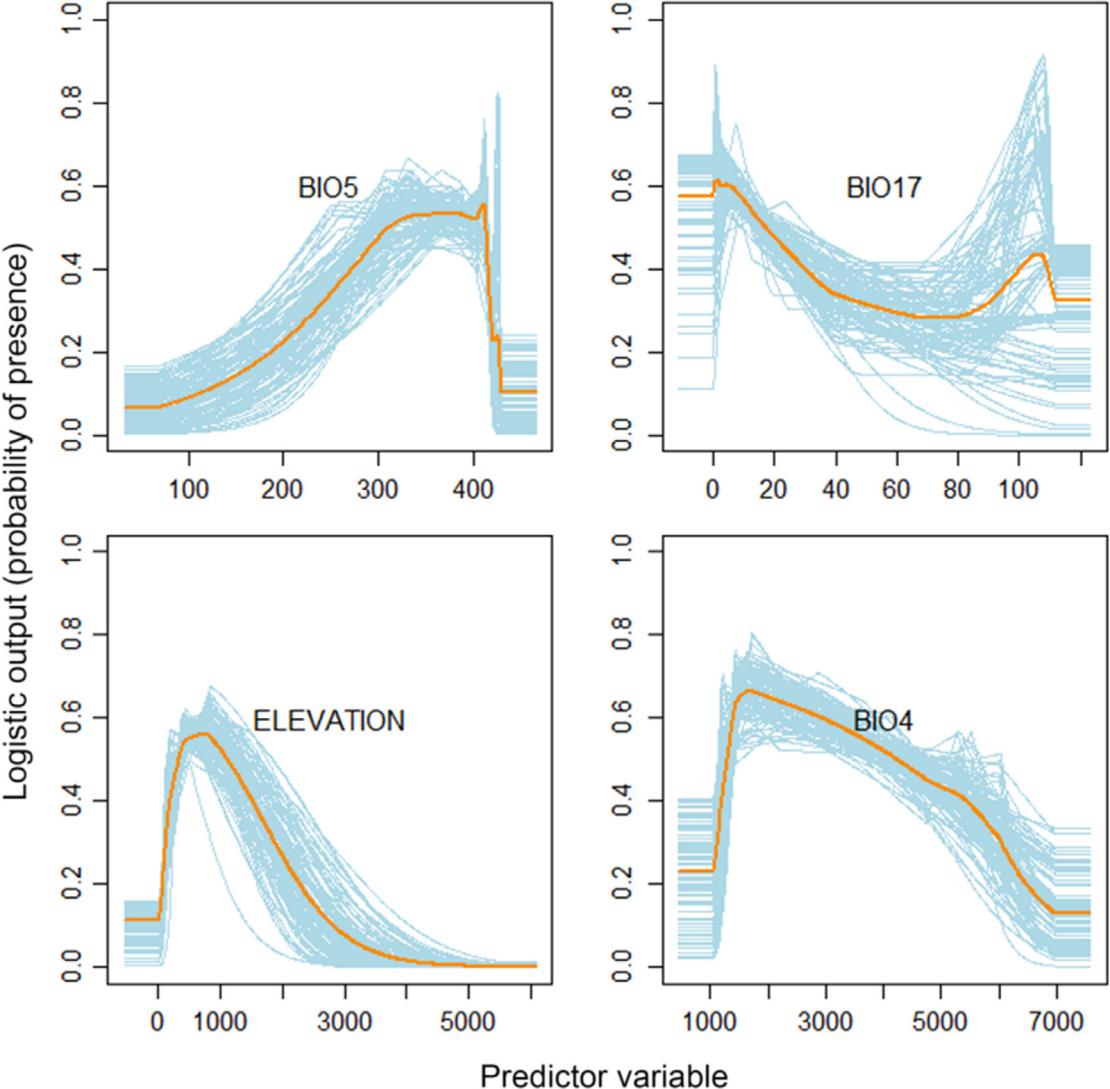
Effects of the four most important variables on MaxEnt prediction of climatic suitability for FHA on the Indian subcontinent. The curves show how the logistic prediction changes when only the corresponding variable is used. These plots reflect the dependence of predicted suitability both on the selected variable and on dependencies induced by correlations between the selected variable and other variables. For variable description, see Table 1.

Maximum temperature of warmest month, precipitation of driest quarter, elevation, and temperature seasonality explained almost half of the models variability (Table 1). As expected, FHA occurrence was positively linked to high temperatures of the warmest month (bio5), corresponding to savannah‐like vegetation. Response curves also show that suitability for the FHA decreased with higher precipitation of driest quarter (bio17), and temperature seasonality (bio4). Moreover, FHA presence probability was highest at about 400–1000 m above sea level and decreased toward lower and higher elevations (Fig. 6).

## Discussion

### Model performance and uncertainty

In our study, we modeled the potential distribution of four‐horned antelope (FHA) throughout India and Nepal with climate, topographic, and land‐use information. Although the sample size of known occurrences was low, results of bootstrapping indicate an acceptable level of model performance and the MaxEnt model predicted a potential FHA distribution range that mainly coincided with the range limits of previous assessments. This is in accordance with other studies, which showed that small sample sizes can provide enough information to predict species distribution ranges over large spatial scale extents (Hernandez et al. 2006; Pearson et al. 2007). In particular, rare species with limited global distribution like FHA can be modeled successfully (Hernandez et al. 2006) due to narrow fundamental niches and thus well‐defined locations along environmental gradients.

MaxEnt by default uses randomly generated background locations and derives information from those locations for model development (Phillips et al. 2006; Mateo et al. 2010). In this case, however, sampling bias can strongly influence model performance and predictions of species distributions (Phillips et al. 2009; Bystriakova et al. 2012; Kramer‐Schadt et al. 2013; Syfert et al. 2013). We accounted for sampling bias and obtained a prediction map that was different from one, which was modeled with random background (Fig. 4). Explicitly, predicted FHA distribution area adhered much closer to observed FHA occurrences in the latter case, while the target group background model prediction was less centered on such occurrences and was able to predict to areas without observations (Fig. 4). As we controlled for sampling bias and restricted the background to a target group, performance of our model was good, whereas allocation of random background would have further increased the AUC.

The maximum entropy approach is one of the best performing methods in predictive species distribution modeling (Elith et al. 2006). However, it may tend to overpredict a species’ range when predictions are made in unknown environmental space. As both, occurrence and target group background records spanned large longitudinal and latitudinal gradients, the predictions of our model were mainly into known environmental space, which increases its credibility.

Presence‐only calibration plots show the fit between predictions and observed occupancy in classes (bins) of similar prediction values. Both models thus displayed reasonable refinement, as demonstrated by the wide range of possible prediction values between zero and one (Fig. 3). However, good calibration was only achieved when we used target group background. The random background model tended to under‐predict FHA habitat, that is, the relative probability of presence was higher than the predicted presence of the species (Fig. 3). This result further supports other results that showed that MaxEnt models performed better when sampling bias had been corrected for (Bystriakova et al. 2012; Kramer‐Schadt et al. 2013; Syfert et al. 2013).

### FHA distribution range

We chose the logistic output for our MaxEnt prediction map, the values of which should be interpreted as relative areas of high and low suitability rather than absolute values of presence probability (c.f. Pearson et al. 2006; Merow et al. 2013). FHA core areas are in the Western Ghats, the central to southern parts of Deccan peninsula (Fig. 4) and the central and eastern parts of the Nepalese Churia range (Fig. 5). It is evident from our models that FHA habitat lies in tropical areas extending from the slopes of the Indian coast up to the Churia range (below ca. 2000 m) as the northernmost limit of its distribution. Another key finding is that areas suitable as habitats of FHA are patchily distributed all over the tropical Indian subcontinent. FHA is a sedentary species (Mallon and Kingswood 2001); therefore, the probability is low that it crosses large matrix areas between fragmented habitat patches. Particularly, the FHA populations in the Churia range might have been isolated from the rest of the population in Indian peninsula because of the large agricultural landscape along the Ganges river basin in‐between, which is predicted to be unsuitable for FHA. Thus, the Gangetic Plain which is known as “bread basket” of South Asia (Aggarwal et al. 2004) represents a geographic barrier for the dispersion of FHA between the Terai Arc and the range in India. As humans dominate the landscape (Persha et al. 2010; Kumar and Yashiro 2014), FHA populations throughout the subcontinent may be exposed to intensive human land use. In particular, the isolated Nepalese FHA population might be at high risk of extinction because of its small distribution range and population size (Johnson 1998; Krishna et al. 2009). Furthermore, eastern parts of Churia range predicted to be suitable as FHA habitat are outside the protected areas and thus might have high poaching pressure. Therefore, in practical, abundance of FHA might be lower in the eastern parts of Churia range than in the western parts. However, more research is needed on dispersal ability of FHA and genetic exchange to understand the viability of its subpopulations, which is estimated to be less than 1,000 individuals (Mallon 2008).

Our prediction map will support ground truthing of FHA occurrence as well as assessment of habitat conditions and protection status of predicted distribution ranges. In particular, our work may assist species management planning including a revision of the conservation status of FHA in Nepal. Moreover, based on our prediction maps and the presence locations from Ahrestani et al. (2011), the distribution range of FHA, as delineated by Leslie and Sharma (2009) and in the IUCN Red List of Threatened Species list (Mallon 2008), may need to be extended further south. Areas in the Gangetic Plain, which were predicted by our model to be least suitable, should be included in ground assessments to identify potential corridors between the Indian and Nepalese populations. Until a revised map has been ground‐checked, our predicted distribution range may provide a valuable basis for conservation management in favor of the FHA and its habitats but also for targeted monitoring. Another interesting feature of our model was its prediction of a small part of Sindh Province in Pakistan as suitable FHA habitat because the species used to be found in those areas (Roberts 1997; Krishna et al. 2009). Reintroduction of FHA in the Sindh Province may prove successful to restore the FHA population.

### Predictors of the FHA distribution

We present the suitable areas for FHA on the Indian subcontinent as explained by topographical and climatic features. We did not directly include land cover information as predictors of the FHA range because there is considerable redundancy between climatic conditions and land cover (Woodward 1987; Woodward et al. 2004). Climate throughout the Indian subcontinent differs considerably among biogeographic zones and results in different land cover and vegetation types (Udvardy 1975; Blasco et al. 1996). Also, inclusion of a multilevel factor variable would have reduced degrees of freedom, which is critical with a small dataset such as the one we used. Rather than land cover, we therefore used climatic variables, which in turn proved to be important as predictors of FHA distribution.

Four‐horned antelope core areas are very dry (7–9 months) and are characterized by undulating terrain with moist and dry deciduous forest, and woodlands (Blasco et al. 1996; Kodandapani et al. 2004; Prasad et al. 2008). Those forest types are subjected to annual wildfires which regulate vegetation structures (Blasco et al. 1996; FAO, 2007). Outputs of our model support these research findings on small‐scale habitat features of FHA, which are mainly determined by tropical dry deciduous forests (Krishna et al. 2008, 2009; Baskaran et al. 2009; Leslie and Sharma 2009; Sharma et al. 2013; Pokharel et al. 2015). The representation of the FHA's distribution in environmental space by higher temperature of warmest months (bio5) and lower precipitation of driest month (bio17) suggests that the species can geographically be found in areas with hot and long dry season. Those areas receive high amounts of rainfall during monsoon. In addition, the curvilinear response to elevation indicates that FHA can be found at intermediate elevations and habitat suitability decreases toward higher elevations above 1,000 m. This relationship of FHA habitat suitability with elevation is evident in our model, which predicted the high Himalayan areas as unsuitable for FHA. Species distribution, however, is indirectly linked to elevation. It is directly connected to the more functional ecological variables such as temperature and precipitation (Elith and Leathwick 2009) which in turn determine vegetation structure and habitat quality. Elevation is also linked to topographical features such as slope and ruggedness, which may affect predation risk. Disregarding the indirect character of elevation, we decided to report FHA's response to this variable as bootstrapped curves showed little variation and the predictor considerably contributed to the model. In addition, an optimum elevation range can be easily measured in the field than averaged climate variables, which facilitates communication to conservation managers.

Temperature affects physiologic processes and hence the behavior of animals (Root 1988; Briffa et al. 2013). Temperature seasonality (bio4) with its negative impact on climatic suitability confirms that fluctuation in temperature throughout the year should not be too high in the FHA's range meaning that climatic habitat suitability for FHA is highest at equatorial region and decreases toward the north and higher elevation. The Churia hills in the Terai Arc, which are characterized by a subtropical climate with mean annual temperature of 20°C and longer dry season (Barnekow Lillesø et al. 2005; MoFSC, 2008), were predicted as the northernmost limit of FHA distribution.

Major river systems of the subcontinent, particularly the Ganges River, are diverted to irrigate almost one‐fourth of the total Ganges river basin. In the agricultural landscape, moisture levels in the air remain high due to evapotranspiration (Thenkabail et al. 2005; Dheeravath et al. 2010). Singh et al. (2008) noticed an increase in summer temperature but a decrease in winter temperature during the last century. Premonsoon rain was also increased leading to a shorter dry season with high seasonality. Thus, human actions have altered the climate of the Ganges basin which might have made the basin climatically unsuitable for FHA.

In conclusion, we presented the first empirical range‐wide distribution model for four‐horned antelope on the Indian subcontinent. Together with the underlying occurrence records, our predictions revealed some suitable areas that are missing from extant range maps but also areas that are obviously unsuitable such as the Gangetic Plains. Areas suitable as habitat for FHA are patchily distributed from the slopes of the Indian coast to the foothills of the Himalaya and are under pressure of human land use. Compared to the whole distribution range of the endemic FHA, its suitable areas in Nepal are very small, fragmented, and isolated from the Indian core distribution. We therefore suggest a revision of the current FHA distribution map as represented in the IUCN Red List of Threatened Species and further actions to protect the species from land‐use changes.

## Conflict of Interest

None declared.

## Supporting information


**Figure S1**. Density curves that visualize the locations of occurrence (green), target group background (blue), and random background (red) values along environmental gradients.

## References

[ece32037-bib-0001] Aggarwal, P. K. , P. K. Joshi , J. S. I. Ingram , and R. K. Gupta . 2004 Adapting food systems of the Indo‐Gangetic plains to global environmental change: key information needs to improve policy formulation. Environ. Sci. Policy 7:487–498.

[ece32037-bib-0002] Ahrestani, F. S. , I. M. A. Heitkönig , F. van Langevelde , S. Vaidyanathan , M. D. Madhusudan , and H. H. T. Prins . 2011 Moisture and nutrients determine the distribution and richness of India's large herbivore species assemblage. Basic Appl. Ecol. 12:634–642.

[ece32037-bib-0003] Anwar, M. , H. Kumar , and J. Vattakavan . 2011 Record of Tetracerus quadricornis (de Blainville, 1816) in Pilibhit Forest division of Terai Arc Landscape, Uttar Pradesh, India. J. Threat. Taxa 3:1719–1721.

[ece32037-bib-0004] Araújo, M. B. , D. Alagador , M. Cabeza , D. Nogués‐Bravo , and W. Thuiller . 2011 Climate change threatens European conservation areas. Ecol. Lett. 14:484–492. 21447141 10.1111/j.1461-0248.2011.01610.x PMC3116148

[ece32037-bib-0005] Barnekow Lillesø, J. P. , T. B. Shrestha , L. P. Dhakal , R. P. Nayaju , and R. Shrestha . 2005 The map of potential vegetation of Nepal: a forestry/agro‐ecological/biodiversity classification system. Center for Skov, Landskab og Planlægning/Københavns Universitet, Copenhagen, Denmark.

[ece32037-bib-0006] Baskaran, N. , A. A. Desai , and A. Udhayan . 2009 Population distribution and conservation of the four‐horned antelope (Tetracerus quadricornis) in the tropical forest of Southern India. J. Sci. Trans. Environ. Technol. 2:139–144.

[ece32037-bib-0007] Blasco, F. , M. F. Bellan , and M. Aizpuru . 1996 A vegetation map of tropical continental Asia at scale 1:5 million. J. Veg. Sci. 7:623–634.

[ece32037-bib-0008] Briffa, M. , D. Bridger , and P. A. Biro . 2013 How does temperature affect behaviour? Multilevel analysis of plasticity, personality and predictability in hermit crabs. Anim. Behav. 86:47–54.

[ece32037-bib-0009] Bystriakova, N. , M. Peregrym , R. H. J. Erkens , O. Bezsmertna , and H. Schneider . 2012 Sampling bias in geographic and environmental space and its effect on the predictive power of species distribution models. Syst. Biodivers. 10:305–315.

[ece32037-bib-0010] Carpenter, C. 2005 The environmental control of plant species density on a Himalayan elevation gradient. J. Biogeogr. 32:999–1018.

[ece32037-bib-0011] Dheeravath, V. , P. S. Thenkabail , G. Chandrakantha , P. Noojipady , G. P. O. Reddy , C. M. Biradar , et al. 2010 Irrigated areas of India derived using MODIS 500 m time series for the years 2001–2003. ISPRS J. Photogramm. Remote Sens. 65:42–59.

[ece32037-bib-0012] Drew, C. A. , Y. Wiersma , and F. Huettmann . 2011 Predictive Species and Habitat Modeling in Landscape Ecology. Springer, New York.

[ece32037-bib-0013] Duan, K. , and T. Yao . 2004 Low‐frequency of southern Asian monsoon variability using a 295‐year record from the Dasuopu ice core in the central Himalayas. Geophys. Res. Lett. 31:L16209.

[ece32037-bib-0014] Elith, J. , and J. R. Leathwick . 2009 Species distribution models: ecological explanation and prediction across space and time. Annu. Rev. Ecol. Evol. Syst. 40:677–697.

[ece32037-bib-0015] Elith, J. , C. Graham , R. Anderson , M. Dudik , H. C. Graham , R. P. Anderson , et al. 2006 Novel methods improve prediction of species’ distributions from occurrence data. Ecography 29:129–151.

[ece32037-bib-0016] Elith, J. , S. J. Phillips , T. Hastie , M. Dudík , Y. E. Chee , and C. J. Yates . 2011 A statistical explanation of MaxEnt for ecologists. Divers. Distrib. 17:43–57.

[ece32037-bib-0017] Ellis, E. C. , K. Klein Goldewijk, , S. Siebert , D. Lightman , and N. Ramankutty . 2010 Anthropogenic transformation of the biomes, 1700 to 2000. Glob. Ecol. Biogeogr. 19:589–606.

[ece32037-bib-0018] FAO . 2007 Fire management: global assessment 2006. FAO Forestry Paper (FAO), Rome, Italy.

[ece32037-bib-0019] Freedman, D. A . 1981 Bootstrapping regression models. Ann. Stat. 9:1218–1228.

[ece32037-bib-0020] Goswami, V. R. , D. Vasudev , and M. K. Oli . 2014 The importance of conflict‐induced mortality for conservation planning in areas of human–elephant co‐occurrence. Biol. Conserv. 176:191–198.

[ece32037-bib-0021] Guisan, A. , and W. Thuiller . 2005 Predicting species distribution: offering more than simple habitat models. Ecol. Lett. 8:993–1009. 10.1111/j.1461-0248.2005.00792.x 34517687

[ece32037-bib-0022] Guisan, A. , and N. E. Zimmermann . 2000 Predictive habitat distribution models in ecology. Ecol. Model. 135:147–186.

[ece32037-bib-0023] Haddad, N. M. , L. A. Brudvig , J. Clobert , K. F. Davies , A. Gonzalez , R. D. Holt , et al. 2015 Habitat fragmentation and its lasting impact on Earth's ecosystems. Sci. Advan. 1:e1500052. 26601154 10.1126/sciadv.1500052 PMC4643828

[ece32037-bib-0024] Hernandez, P. A. , C. H. Graham , L. L. Master , and D. L. Albert . 2006 The effect of sample size and species characteristics on performance of different species distribution modeling methods. Ecography 29:773–785.

[ece32037-bib-0025] Hijmans, R. J. , S. E. Cameron , J. L. Parra , P. G. Jones , and A. Jarvis . 2005 Very high resolution interpolated climate surfaces for global land areas. Int. J. Climatol. 25:1965–1978.

[ece32037-bib-0026] Hijmans, R. J. , S. Phillips , J. Leathwick , and J. Elith . 2015 dismo: Species distribution modeling. R package version 1.0‐12, http://CRAN.R-project.org/package=dismo.

[ece32037-bib-0027] IUCN & UNEP‐WCMC . 2015 The World database on protected areas [Online], November/2015. World Wide Web electronic Publication, www.protectedplanet.com.

[ece32037-bib-0028] Jnawali, S. , H. Baral , S. Lee , K. Acharya , G. Upadhyay , M. Pandey , et al. 2011 The status of Nepal's mammals: The National Red List Series. Department of National Parks and Wildlife Conservation, Kathmandu, Nepal.

[ece32037-bib-0029] Johnson, D. 1980 The comparison of usage and availability measurements for evaluating resource preference. Ecology 61:65–71.

[ece32037-bib-0030] Johnson, C. N. 1998 Species extinction and the relationship between distribution and abundance. Nature 394:272–274.

[ece32037-bib-0031] Joseph, S. , K. Anitha , and M. S. R. Murthy . 2009 Forest fire in India: a review of the knowledge base. J. For. Res. 14:127–134.

[ece32037-bib-0032] Karanth, K. , and M. Sunquist . 1992 Population structure, density and biomass of large herbivores in the tropical forests of Nagarahole, India. J. Trop. Ecol. 8:21–35.

[ece32037-bib-0033] Kodandapani, N. , M. A. Cochrane , and R. Sukumar . 2004 Conservation threat of increasing fire frequencies in the Western Ghats, India. Conserv. Biol. 18:1553–1561.

[ece32037-bib-0034] Kramer‐Schadt, S. , J. Niedballa , J. D. Pilgrim , B. Schröder , J. Lindenborn , V. Reinfelder , et al. 2013 The importance of correcting for sampling bias in MaxEnt species distribution models. Divers. Distrib. 19:1366–1379.

[ece32037-bib-0035] Krishna, Y. C. Y. , J. Krishnaswamy , and N. S. Kumar . 2008 Habitat factors affecting site occupancy and relative abundance of four‐horned antelope. J. Zool. 276:63–70.

[ece32037-bib-0036] Krishna, Y. C. , P. J. Clyne , J. Krishnaswamy , and N. S. Kumar . 2009 Distributional and ecological review of the four horned antelope, Tetracerus quadricornis. Mammalia 73:1–6.

[ece32037-bib-0037] Kumar, P. , and M. Yashiro . 2014 The marginal poor and their dependence on ecosystem services: evidence from South Asia and Sub‐Saharan Africa Pp. 169–180 *in* von Braun J., and Gatzweiler F. W., eds. Marginality. Springer, the Netherlands.

[ece32037-bib-0038] Leslie, D. M. , and K. Sharma . 2009 Tetracerus quadricornis (Artiodactyla: Bovidae). Mamm. Spec. 843:1–11.

[ece32037-bib-0039] Mallon, D . 2008 Tetracerus quadricornis. IUCN Red List of Threatened Species. Version 2014.2.

[ece32037-bib-0040] Mallon, D. , and S. Kingswood . 2001 Antelopes. Part 4. North Africa, the Middle East, and Asia. Global survey and regional action plans. SSC Antelope Specialist Group, IUCN, Gland and Cambridge.

[ece32037-bib-0041] Mateo, R. G. , T. B. Croat , Á. M. Felicísimo , and J. Muñoz . 2010 Profile or group discriminative techniques? Generating reliable species distribution models using pseudo‐absences and target‐group absences from natural history collections. Divers. Distrib. 16:84–94.

[ece32037-bib-0042] Mathys, L. , N. E. Zimmermann , N. Zbinden , and W. Suter . 2006 Identifying habitat suitability for hazel grouse Bonasa bonasia at the landscape scale. Wild. Biol. 12:357–366.

[ece32037-bib-0043] Merow, C. , M. J. Smith , and J. A. Silander . 2013 A practical guide to MaxEnt for modeling species’ distributions: what it does, and why inputs and settings matter. Ecography 36:1058–1069.

[ece32037-bib-0044] Mishra, C. 1997 Livestock depredation by large carnivores in the Indian trans‐Himalaya: conflict perceptions and conservation prospects. Environ. Conserv. 24:338–343.

[ece32037-bib-0045] MoFSC . 2008 Churia Area Program Strategy. 55.

[ece32037-bib-0046] Mooley, D. A. , and B. Parthasarathy . 1983 Variability of the Indian summer monsoon and tropical circulation features. Mon. Weather Rev. 111:967–978.

[ece32037-bib-0047] Parthasarathy, B. , A. A. Munot , and D. R. Kothawale . 1994 All‐India monthly and seasonal rainfall series: 1871–1993. Theoret. Appl. Climatol. 49:217–224.

[ece32037-bib-0048] Pearson, R. G. , W. Thuiller , M. B. Araújo , E. Martinez‐Meyer , L. Brotons , C. McClean , et al. 2006 Model‐based uncertainty in species range prediction. J. Biogeogr. 33:1704–1711.

[ece32037-bib-0049] Pearson, R. G. , C. J. Raxworthy , M. Nakamura , and P. A. Townsend . 2007 Predicting species distributions from small numbers of occurrence records: a test case using cryptic geckos in Madagascar. J. Biogeogr. 34:102–117.

[ece32037-bib-0050] Persha, L. , H. Fischer , A. Chhatre , A. Agrawal , and C. Benson . 2010 Biodiversity conservation and livelihoods in human‐dominated landscapes: Forest commons in South Asia. Biol. Conserv. 143:2918–2925.

[ece32037-bib-0051] Phillips, S. J. , and J. Elith . 2010 POC plots: calibrating species distribution models with presence‐only data. Ecology 91:2476–2484. 20836469 10.1890/09-0760.1

[ece32037-bib-0052] Phillips, S. J. , R. P. Anderson , and R. E. Schapire . 2006 Maximum entropy modeling of species geographic distributions. Ecol. Model. 190:231–259.

[ece32037-bib-0053] Phillips, S. J. , M. Dudík , J. Elith , C. H. Graham , A. Lehmann , J. Leathwick , et al. 2009 Sample selection bias and presence‐only distribution models: implications for background and pseudo‐absence data. Ecol. Appl. 19:181–197. 19323182 10.1890/07-2153.1

[ece32037-bib-0054] Pokharel, K. P. , T. Ludwig , and I. Storch . 2015 Spatial niche partitioning in sub‐tropical solitary ungulates: four‐horned antelope and barking deer in Nepal. PLoS One 10:e0117917. 25714092 10.1371/journal.pone.0117917 PMC4340944

[ece32037-bib-0055] Porwal, M. C. , P. S. Roy , and V. Chellamuthu . 1996 Wildlife habitat analysis for “sambar” (Cervus unicolor) in Kanha National Park using remote sensing. Int. J. Remote Sens. 17:2683–2697.

[ece32037-bib-0056] Prasad, V. K. , K. V. S. Badarinath , and A. Eaturu . 2008 Biophysical and anthropogenic controls of forest fires in the Deccan Plateau, India. J. Environ. Manage. 86:1–13. 17275159 10.1016/j.jenvman.2006.11.017

[ece32037-bib-0057] Puyravaud, J.‐P. , P. Davidar , and W. F. Laurance . 2010 Cryptic destruction of India's native forests. Conserv. Lett. 3:390–394.

[ece32037-bib-0058] R Core Team . 2013 R: A language and environment for statistical computing. Vienna, Austria URL http://www.R-project.org/.

[ece32037-bib-0059] Roberts, T. J . 1997 The mammals of Pakistan. Oxford University Press, London.

[ece32037-bib-0060] Root, T. 1988 Energy constraints on Avian distribution and abundances. Ecology 69:330–339.

[ece32037-bib-0061] RStudio Team . 2015 RStudio: Integrated development for R. RStudio Inc, Boston, MA.

[ece32037-bib-0062] Sanderson, E. W. , K. H. Redford , A. Vedder , P. B. Coppolillo , and S. E. Ward . 2002 A conceptual model for conservation planning based on landscape species requirements. Land. Urban Plann. 58:41–56.

[ece32037-bib-0063] Sekhar, N. 1998 Crop and livestock depredation caused by wild animals in protected areas: the case of Sariska Tiger Reserve, Rajasthan, India. Environ. Conserv. 25:160–171.

[ece32037-bib-0064] Sharma, K. , R. S. Chundawat , J. Van Gruisen , and A. R. Rahmani . 2013 Understanding the patchy distribution of four‐horned antelope Tetracerus quadricornis in a tropical dry deciduous forest in Central India. J. Trop. Ecol. 30:45–54.

[ece32037-bib-0065] Shrestha, M. L. 2000 Interannual variation of summer monsoon rainfall over Nepal and its relation to Southern Oscillation Index. Meteorol. Atmos. Phys. 75:21–28.

[ece32037-bib-0066] Shrestha, A. B. , C. P. Wake , J. E. Dibb , and P. A. Mayewski . 2000 Precipitation fluctuations in the Nepal Himalaya and its vicinity and relationship with some large scale climatological parameters. Int. J. Climatol. 20:317–327.

[ece32037-bib-0067] Singh, P. , V. Kumar , T. Thomas , and M. Arora . 2008 Changes in rainfall and relative humidity in river basins in northwest and central India. Hydrol. Process. 22:2982–2992.

[ece32037-bib-0068] Southworth, J. , H. Nagendra , and L. Cassidy . 2012 Forest transition pathways in Asia – studies from Nepal, India, Thailand, and Cambodia. J. Land Use Sci. 7:51–65.

[ece32037-bib-0069] Syfert, M. M. , M. J. Smith , and D. A. Coomes . 2013 The effects of sampling bias and model complexity on the predictive performance of MaxEnt species distribution models. PLoS One 8:e55158. 23457462 10.1371/journal.pone.0055158 PMC3573023

[ece32037-bib-0070] Thenkabail, P. S. , M. Schull , and H. Turral . 2005 Ganges and Indus river basin land use/land cover (LULC) and irrigated area mapping using continuous streams of MODIS data. Remote Sens. Environ. 95:317–341.

[ece32037-bib-0501] USGS 2006, Shuttle Radar Topography Mission, 30 Arc Second scene (tile 28), Unfilled Unfinished 2.0, Global Land Cover Facility, University of Maryland, College Park, Maryland, February 2000.

[ece32037-bib-0071] Udvardy, M. D. F . 1975 A classification of the biogeographical provinces of the world. Switzerland: IUCN Occasional Paper 18. IUCN, Morges, Switzerland.

[ece32037-bib-0072] WCU‐UNEP . 2007 World database on protected areas, version 2007. Cambridge: United Nations Environment Programme‐World Conservation Monitoring Centre.

[ece32037-bib-0073] Woodward, F. I. 1987 Climate and plant distribution. Cambridge University Press, Cambridge, Cambridge.

[ece32037-bib-0074] Woodward, F. I. , M. R. Lomas , and C. K. Kelly . 2004 Global climate and the distribution of plant biomes. Philos. Trans. Royal Soc. London Series B, Biol. Sci. 359:1465–1476. 10.1098/rstb.2004.1525 PMC1693431 15519965

