# Supplementary figures and images for: Predicting potential distribution of poorly known species with small database: the case of four‐horned antelope Tetracerus quadricornis on the Indian subcontinent
Source: Ecol Evol. 2016 Mar 4;6(8):2297–307. doi: 10.1002/ece3.2037 (PMC4782261; doi:10.1002/ece3.2037)

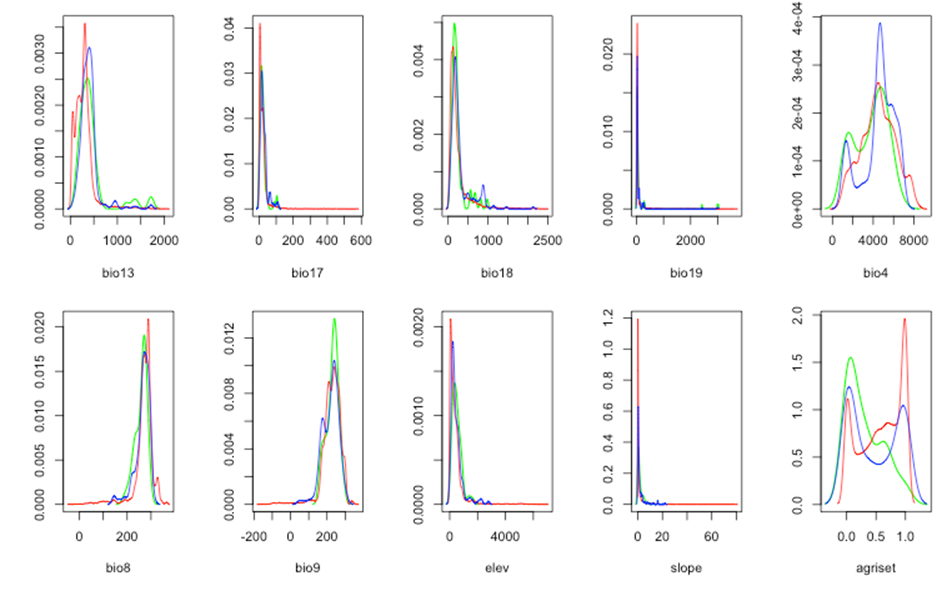

Supplement: Supplementary file 1 — Figure S1. Density curves that visualize the locations of occurrence (green), target group background (blue), and random background (red) values along environmental gradients. [file ECE3-6-2297-s001.tiff]
